# Expression analysis of the mouse S100A7/psoriasin gene in skin inflammation and mammary tumorigenesis

**DOI:** 10.1186/1471-2407-5-17

**Published:** 2005-02-17

**Authors:** Meghan Webb, Ethan D Emberley, Michael Lizardo, Salem Alowami, Gefei Qing, Abdullah Alfia'ar, Linda J Snell-Curtis, Yulian Niu, Alberto Civetta, Yvonne Myal, Robert Shiu, Leigh C Murphy, Peter H Watson

**Affiliations:** 1 Manitoba Institute of Cell Biology, University of Manitoba, Winnipeg, Canada; 2 Dept of Physiology, University of Manitoba, Winnipeg, Canada; 3 Dept of Pathology, University of Manitoba, Winnipeg, Canada; 4 Dept of Biochemistry & Medical Genetics, University of Manitoba, Winnipeg, Canada; 5 Dept of Biology, University of Winnipeg, Winnipeg, Canada

## Abstract

**Background:**

The human psoriasin (S100A7) gene has been implicated in inflammation and tumor progression. Implementation of a mouse model would facilitate further investigation of its function, however little is known of the murine psoriasin gene. In this study we have cloned the cDNA and characterized the expression of the potential murine ortholog of human S100A7/psoriasin in skin inflammation and mammary tumorigenesis.

**Methods:**

On the basis of chromosomal location, phylogenetic analysis, amino acid sequence similarity, conservation of a putative Jab1-binding motif, and similarities of the patterns of mouse S100A7/psoriasin gene expression (measured by RT-PCR and in-situ hybridization) with those of human S100A7/psoriasin, we propose that mouse S100A7/psoriasin is the murine ortholog of human psoriasin/S100A7.

**Results:**

Although mouse S100A7/psoriasin is poorly conserved relative to other S100 family members, its pattern of expression parallels that of the human psoriasin gene. In murine skin S100A7/psoriasin was significantly upregulated in relation to inflammation. In murine mammary gland expression is also upregulated in mammary tumors, where it is localized to areas of squamous differentiation. This mirrors the context of expression in human tumor types where both squamous and glandular differentiation occur, including cervical and lung carcinomas. Additionally, mouse S100A7/psoriasin possesses a putative Jab1 binding motif that mediates many downstream functions of the human S100A7 gene.

**Conclusion:**

These observations and results support the hypothesis that the mouse S100A7 gene is structurally and functionally similar to human S100A7 and may offer a relevant model system for studying its normal biological function and putative role in tumor progression.

## Background

Human S100A7 or psoriasin was first identified as an over-expressed secreted protein in psoriatic skin[1]. More recently its expression in both pre-invasive (ductal carcinoma *in situ*, DCIS) and invasive human breast cancer was demonstrated [2]. Although highly expressed in DCIS and generally down-regulated in invasive breast cancer, the expression of psoriasin/S100A7 in both in-situ and invasive breast cancer is correlated with markers of poor prognosis [3,4] and in invasive carcinoma also with poor clinical outcome [5]. Support for psoriasin/S100A7 having a functional role in this aggressive phenotype is shown by the observation of increased growth and tumorigenesis when breast cancer cells over-expressing psoriasin/S100A7 are grown as xenografts in nude mice [6]. This activity may in part be mediated by the ability of psoriasin/S100A7 to interact with c-Jun activation domain-binding protein 1 (Jab1) [6] and enhance pro-survival pathways [7] and protect against anoikis in human breast and head and neck squamous cancer cells [8]. However, further investigation of the physiological and pathophysiological function of psoriasin/S100A7 can only be effectively undertaken using genetic manipulation of an animal model. To undertake such studies we need to characterize and clone the mouse psoriasin gene. Interestingly, during the preparation of this manuscript Marenholz *etal.*, [9] have argued that the possible ancestral homolog of the human S100A7 paralogs [10] has been identified in the mouse on the basis of genome sequence analysis and gene orientation, but has not been characterized fully as yet. The following study provides evidence of the cloning of a cDNA of the putative mouse ortholog of human psoriasin/S100A7, investigates its expression under conditions of mouse mammary tumorigenesis and skin inflammation, and confirms that the factors involved in its regulation are comparable to those involved in regulation of the human counterpart.

## Methods

### Tissues

Murine studies were conducted in accordance with the principles and procedures recommended and approved by the University of Manitoba Animal Care Review Board. Mammary tumors in CD1 mice were generated chemically using 7, 12-dimethylbenz anthracine (DMBA) as previously described [11]. Freshly dissected mouse tissues were either stored frozen at -70°C, or processed to generate formalin fixed paraffin-embedded tissue blocks.

Acute dermatitis in C57/B6 mice was induced by the topical application of 20% croton oil (dissolved in dimethyl sulfoxide) to a 1 cm length mid-tail portion [12]. The tail skins were stimulated continuously with croton oil every 4 hours for 24 hours. Mice were divided into five time-groups (each containing 3 mice) that were designated 0, 4, 8, 16, and 24 hours. At each time-point, the mice were sacrificed. The tail-skins were harvested and paired specimens of normal and inflamed skin tissue were fixed in 3.7% formaldehyde in 0.1 M phosphate buffered saline for 16–18 hours, followed by paraffin embedding.

Sections from the above blocks were used for preparation of haematoxylin and eosin stained sections for light microscopic examination, *in situ *hybridization (ISH), and immunohistochemistry (IHC).

Human tumor tissues were obtained from the Department of Pathology, University of Manitoba. All cases were coded and therefore anonymous and prior approval was obtained from the University of Manitoba Research Ethics Board and the Pathology Access to Tissue Committee. Different cervical tumor pathologies (total n = 39 cases) including in-situ adenocarcinoma (n = 10) and squamous cell carcinoma (n = 9), as well as invasive adenocarcinoma (n = 10) and squamous carcinoma (n = 10) were selected for one cohort study. In addition, different invasive lung tumor types (total n = 78 cases) including mesothelioma (n = 10), small cell carcinoma (n = 15), adenocarcinoma (n = 28), and squamous carcinoma (n = 25) were selected to form another study cohort. Squamous differentiation within both murine and human tumors was determined by standard morphological criteria including cytoplasmic keratinization and cellular stratification relative to keratin pearls.

### In-situ hybridization

Paraffin embedded 5 μm tissue sections were analyzed by *in-situ *hybridization according to a previously described protocol [13]. The plasmid pCR4-TOPO-mPsor-ORF, consisted of pCR4-TOPO plasmid (Invitrogen Canada Inc, Burlington, ON) containing a 344 base pair insert of the mouse psoriasin cDNA, also known as mouse S100A15 (from nucleotide 94 to 437 as numbered in AY465109, and from 1 to 344 as numbered in AY582964). One microgram of linearized template DNA was used to generate ^35^S-UTP-labeled sense and antisense cRNA probes using the Riboprobe System (Promega, Madison, WI) according to the manufacturer's instructions. Sense probes were used as controls. In-situ hybridization and washing conditions were as previously described[2]. Sections were developed using Kodak NTB-2 photographic emulsion and counter-stained with Lee's stain after 2–6 weeks.

Levels of mouse S100A7/psoriasin RNA expression were assessed by microscopic examination at low power magnification and with reference to the negative sense control. This was done by scoring the estimated average signal intensity (on a scale of 0 to 3), where 0 is no expression and 3 is a high proportion of strong focal expression.

### Immunohistochemistry

Immunohistochemistry (IHC) was performed on serial 5 μm sections from a representative, formalin fixed paraffin embedded tissue block from each tumor. For human tumor blocks, psoriasin/S100A7 IHC was performed essentially as described [5] and human psoriasin/S100A7 was detected using a previsouly characterized rabbit polyclonal antibody [3,5]. For murine tumors estrogen receptor-alpha (ERα) was detected using an affinity purified rabbit polyclonal antibody, MC-20, raised against a C-terminal peptide of mouse ERα (#sc-542, Santa Cruz Biotechnology Inc, CA). Antibodies were applied using an automated tissue immunostainer (Discovery module, Ventana Medical System), 3, 3-diaminobenzidine IHC kit and bulk reagents were supplied by the manufacturer. Briefly, the Discovery staining protocol was set to "Standard Cell Conditioning", followed by 60 minutes incubation at 42°C with primary antibody and 30 minutes incubation at 42°C with secondary antibody (goat anti-rabbit-IgG-HRP, Jackson Immuno Research Labs Inc). Primary antibody concentrations initially applied to the Ventana instrument were 1:200 for ERα and 1:200 for the secondary antibody translating into final dilutions of 1:600 after 1:3 dilution with buffer dispensed onto the slide with the primary antibody. Slides were counterstained with hematoxylin.

Levels of psoriasin/S100A7 and ERα expression were scored semi-quantitatively in tissue sections, under the light microscope. Scores were obtained by estimating average signal intensity (scale of 0 to 3) and the proportion of epithelial cells showing a positive signal (0–100%). The intensity and proportion scores were then multiplied to give an overall IHC-score.

### RNA extraction and reverse transcription

Total mouse RNA was extracted using Trizol™ reagent (Invitrogen) according to the manufacturer's instructions, and the integrity of the RNA was confirmed by denaturing gel electrophoresis as previously described [14]. RNAs from the various frozen tissues were reverse transcribed. One μg of total RNA was reverse transcribed in a final volume of 30 μl composed of 50 mM Tris-HC1 (pH 8.3), 75 mM KC1, 3 mM MgC1_2_, 0.5 μM random hexamers (Invitrogen) 0.5 mM dNTPs, 0.01 mM DTT in the presence of 300 units of MMLV-RT (Invitrogen), and 4 units RNase inhibitor at 37°C for 1 hour, followed by 5 minutes at 95°C and kept at -20°C until used.

### PCR conditions

The primer pairs used were as follows;

#### Mouse S100A7/psoriasin C-terminus

5'-ATG CCA GAC ACA CCA GTG GAG-3' (sense; nucleotides 111–131 in GenBank acc. AY465109) and 5'-GGT AGT CCT TCA CCA GCT TGC-3' (antisense; nucleotides 358–378).

#### Mouse S100A7/psoriasin open reading frame

5'-TGA AGG GTC CAT CAG TCA-3' (sense; nucleotides 94–111 in GenBank acc. AY465109) and 5'-CTA GTA GAG GCT GTG CT-3' (antisense; nucleotides 421–437).

#### Mouse β-actin

Primers were designed according to the mRNA sequence (GenBank acc. NM_007393): 5'-TCT ACG AGG GCT ATG CTC TCC-3' (sense; nucleotides 574–594) and 5'-GGA TGC CAC AGG ATT CCA TAC-3' (antisense; nucleotides 883–903). According to the chromosome 5 genomic contig sequence (GenBank acc. NT_039324), these primers span an 87-bp intron with the antisense primer binding across the intron-exon boundary.

PCR reactions were performed essentially as previously described [3]. To amplify cDNA corresponding to mouse S100A7/psoriasin, an initial 2 minutes at 94°C was followed by 36 cycles (30 seconds at 94°C, 30 seconds at 56°C, 30 seconds at 72°C). Twenty six cycles were used to amplify β-actin cDNA (30 seconds at 94°C, 30 seconds at 58°C, 30 seconds at 72°C). PCR products were separated on 1.5% agarose gels containing ethidium bromide (0.1 μg/ml) as previously described [13]. Identity of the 344 bp product corresponding to mouse S100A7/psoriasin and the 330 bp product corresponding to β-actin were confirmed by subcloning and sequencing as described previously [13].

Semi-quantitative PCR analyses were performed using three independent PCRs for each sample for both mouse S100A7/psoriasin and β-actin. Signals visualized with UV irradiation on a GelDoc2000/ChemiDoc System (Bio-Rad), were quantified by densitometry using the Quantity One software (version 4.2; Bio-Rad). Mouse S100A7/psoriasin expression was standardized to β-actin expression assessed from the same cDNA in separate PCR reactions and run in parallel on separate gels. The standardized mean of each triplicate PCR was then expressed relative to the levels in a "moderately expressing" sample selected for each batch of cDNAs to be analyzed.

### Phylogenetic analysis

Amino acid sequences of S100 family genes were aligned using Clustal X [15]. This alignment was used to construct a phylogenetic tree based on a Poisson corrected neighbour-joining distance method [16] available in the computer software package MEGA v3.0 [17]. The reliability of the phylogeny's interior branches was tested by a bootstrap test with 1000 replications [18]. The human sequences used, were GenBank Accession numbers: AAH05019 (S100A14), NP_789793 (5100A15), NP_006262 (S100A1), NP_005969 (S100A2), NP_002951 (S100A3), AAH00838 (S100A4), NP_002953 (S100A5), AAH09017 (S100A6), AAH34687 (S100A7), XP_060509 (S100A7L-2), AAH05928 (S100A8), AAH47681 (S100A9), AAH15973 (S100A10), AAH14354 (S100A11), NP_005612 (S100A12), NP_002952 (S100A13). The mouse sequences used, were GenBank Accession numbers: NP_035439 (S100A1), NP_035440 (S100A3), NP_035441 (S100A4), NP_035442 (S100A5), AAH03832 (S100A6), NP_955454 (S100A7/15), NP_038678 (S100A8), AAH27635 (S100A9), AAH25044 (S100A10), AAH21916 (S100A11), NP_033139 (S100A13), AAH25607 (S100A14).

## Results

### Identification and cloning of mouse S100A7/psoriasin cDNA

**Figure 1:**
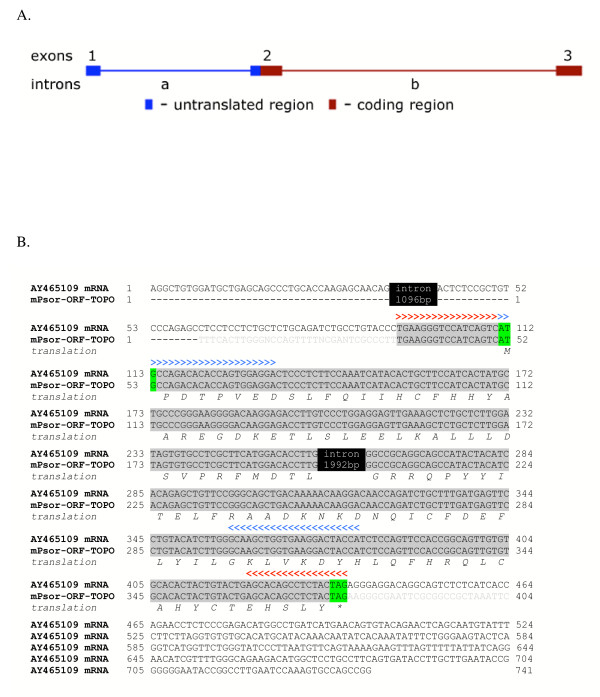
Cloned mouse S100A7/psoriasin sequence, and gene structure A The mouse S100A7 gene sequence (GenBank acc. AY465110) describes a 3829 bp gene with 3 exons and 2 introns. B The sequence of our mPsor-ORF-TOPO clone isolated by RT-PCR of RNA from a DMBA mouse mammary tumor is shown compared with the mouse S100A15 cDNA sequence (GenBank acc. AY465109) isolated from mouse skin. Predicted introns are inserted into the cDNA sequences (black), the predicted open reading frame (ORF) and translated products (amino acid one letter code in italics below the nucleotide sequence) are shown with start and stop codons (green). Vector sequences of the mPsor-ORF-TOPO are indicated in light grey. Sense (>) and antisense (<) primer annealing sites are indicated for both primer sets: mouse psoriasin (blue) and mouse psoriasin-ORF (red).

**Figure 2:**
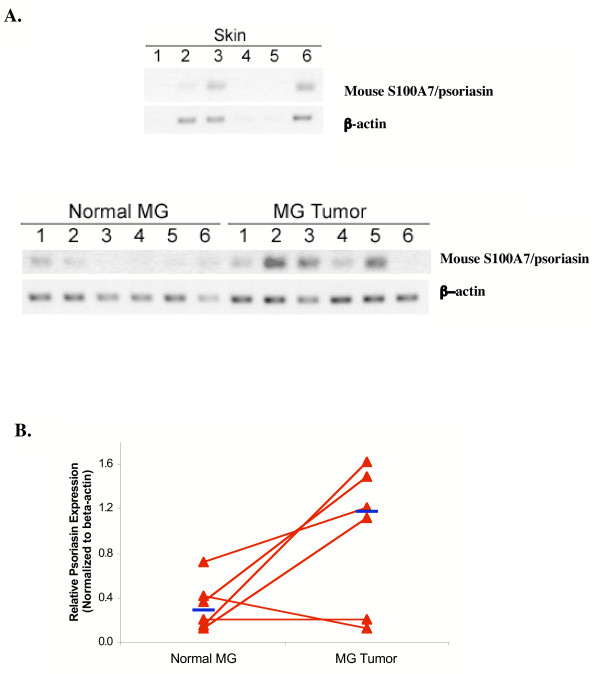
A Top panel Expression of mouse S100A7/psoriasin in normal skin RNA from normal mouse skin from 6 different animals (1–6) was subjected to RT-PCR analysis using mouse psoriasin-ORF primers and β-actin primers as described in Materials and Methods. PCR products of 344 bp for mouse S100A7/psoriasin and 330 bp for β-actin are shown. Bottom panel Expression of mouse S100A7/psoriasin in normal mouse mammary gland) and DMB A induced mammary tumors RNA from freshly frozen matched normal mammary gland (Normal MG) and DMBA induced mammary tumors (MG tumor) from 6 different CD1 mice (1–6) was extracted and subjected to RT-PCR analysis using mouse psoriasin-ORF primers and β-actin primers as described in Materials and Methods. PCR products of 344 bp for mouse S100A7/psoriasin and 330 bp for β-actin are shown. B Semi-quantitative analysis of the results shown in bottom panel of A Data points represent the means of three independent PCR reactions. The value of mouse S100A7 normalized to β-actin for each matched normal MG and MG tumor sample are joined by red lines. Blue lines represent the median value of mouse S100A7/psoriasin expression for each tissue type. Increased expression in MG tumors is significant (p < 0.05, one sided Wilcoxon signed rank test).

**Figure 3:**
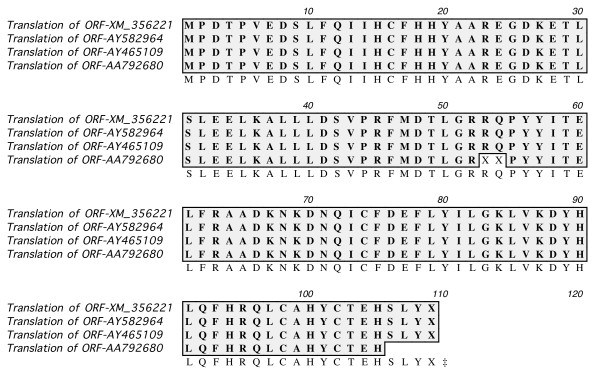
Alignment of the translation products of the predicted ORFs from the various GenBank entries related to mouse S100A7/psoriasin.

A BLASTP search of mouse sequence databases was performed using the human psoriasin (S100A7) amino acid sequence and identified a mouse sequence highly similar to human S100A7, which had been previously predicted by automated computational analysis (GenBank acc. XM_143311, later replaced by AY465109). Using this predicted sequence in a BLASTN search, a mouse skin expressed sequence tag (EST) (GenBank acc. AA792680) containing a highly similar sequence was identified. A pair of mouse "psoriasin"-specific PCR primers was designed that would only amplify a sequence of mouse psoriasin contained within the EST. According to the recently posted genomic sequence (GenBank acc. AY465110) these primers span a 1992-bp intron, thus the potential amplification of contaminating genomic DNA is minimized (Figure 1). An additional set of mouse psoriasin primers ("psoriasin-ORF") was later designed to amplify the entire predicted open reading frame (Figure 1). These primers also span the 1992-bp intron. Both sets of putative mouse psoriasin primers were used for RT-PCR and semi-quantitatively measure the potential expression of the putative mouse psoriasin in mouse skin, mammary gland and mouse mammary tumors. These tissues were chosen because the human psoriasin/S100A7 was described as differentially expressed in skin and breast. The results, using primers that amplified the entire predicted ORF of the putative mouse psoriasin, are shown in Figure 2, with β-actin as a control in parallel. A 344 bp PCR product was amplified from cDNA derived from normal mouse skin and mammary gland using mouse psoriasin primers (see above for description). Based on our PCR results, mouse psoriasin mRNA is expressed in skin as well as in mammary gland where it is more highly expressed in mammary tumors than in normal mammary gland (Figure 2a). The psoriasin RT-PCR product and the 330 bp product generated using the mouse β-actin primers were both cloned and sequenced to confirm their identities. The nucleotide sequence of the putative mouse psoriasin RT-PCR product (GenBank acc. AY582964) was 100% identical to the coding sequence of the putative mRNA in the GenBank sequence database (GenBank acc. XM_143311). Start/stop codons, PCR primer binding sites, and intron/exon boundaries are indicated in Figure 1. However, during the course of this study the predicted mRNA sequence of GenBank acc. XM_143311, named as 'similar to the S100 calcium-binding protein A7 (psoriasin)' was removed and apparently replaced by another entry GenBank acc. XM_356221 for a predicted mRNA that was named as 'similar to the S100 calcium-binding proteins A15'. GenBank acc. AY465109 then appeared as a cDNA cloned from mouse skin and was named as 'Mus musculus S100 calcium-binding protein A15 mRNA'. The amino acid sequences of the various Genbank entries believed to describe the mouse psoriasin are aligned in Figure 3 to illustrate that the amino acid sequences of the ORF from each entry are identical. From here on, we will refer to this gene as mouse S100A7/psoriasin. In addition, according to the recently posted genomic sequence (GenBank acc. AY465110 and NT_078386), the mouse S100A7/psoriasin gene maps within the murine S100 cluster on chromosome 3 (LOC381493, NCBI Map Viewer Link), in a region similar to the human S100 cluster on chromosome 1q21 [10,19]. Our sequence database searches and RT-PCR experiments demonstrate a putative mouse ortholog of the human psoriasin gene exists and like human psoriasin, it is expressed in skin and mammary tissue.

### Phylogenetic analysis

**Figure 4:**
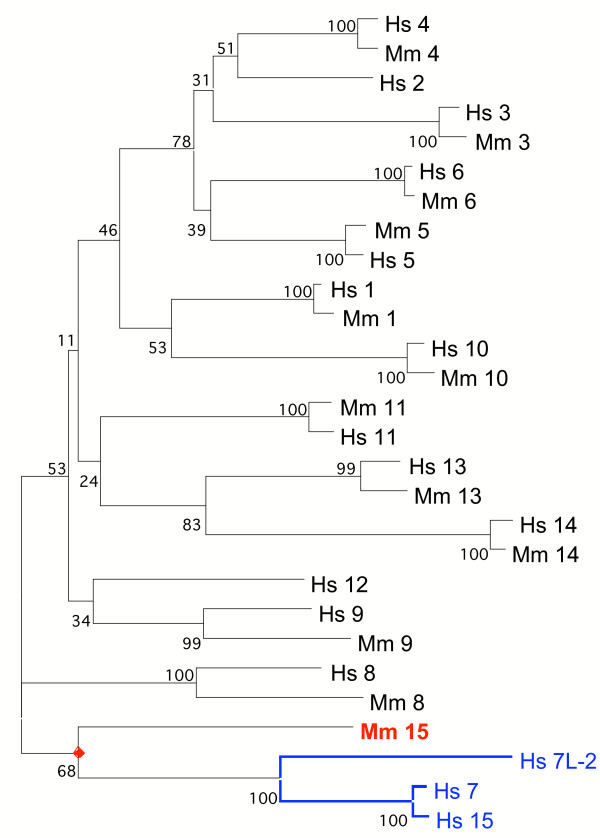
Phylogenetic analysis of human and mouse S100 proteins A neighbour-joining tree of human (Hs) and mouse (Mm) S100 proteins (1 through 15) was constructed using the Poisson correction model and the bootstrap percentage from 1000 replicates is indicated at each node. Results are consistent with the human S100A7 duplications (blue sub-tree) occurring after the divergence of mouse and human in evolution (red node).

The putative mouse psoriasin gene (originally "Similar to S100A7") has been renamed "mouse S100A15" in Genbank (AY465110) [20]. This name implies higher homology to human S100A15 than to human psoriasin/S100A7. However, it has been shown that human S100A7 and S100A15 diverged sometime during primate evolution [10]. A phylogenetic tree was constructed to examine the evolutionary relationships between the mouse and human S100 genes (Figure 4). The results are consistent with the divergence of human S100A7 and S100A15 occurring by duplication after the human/mouse split in evolution, and thus the mouse gene is likely ancestral to both human paralogs.

### Presence of putative Jab1 binding motif in mouse S100A7/psoriasin

A notable structural feature of the human psoriasin/S100A7 gene is a consensus Jab1-binding domain sequence that we have speculated may confer the ability of human psoriasin/S100A7 to interact with Jab1 and so play an important role in the mechanism by which human psoriasin/S100A7 medicates its action [6]. Jab1 was originally identified as a factor influencing c-Jun transcription of AP-1 regulated genes [21]. It is now known that Jab1 is a component of a multimeric protein complex (the CSN/COP9 signalosome) and that Jab1 interacts with many components of cell signalling pathways [22]. This may be in the context of either phosphorylation or proteasomal activities as Jab1 is also the only known deneddylating protein active in control of the SCF-cullin ubiquitin ligases [23].

**Figure 5:**
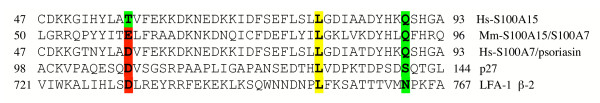
Conservation of the Jab1 binding motif in human psoriasin/S100A7 and mouse S100A7/psoriasin Human psoriasin/S100A7 contains a Jab1 binding domain identified previously in p27 and LFA-1. Mouse S100A7/psoriasin also contains the Jab1 binding motif but human S100A15 does not. Note that human S100A15 has a significant change, from a charged (D) to a neutral (T) amino acid, in the first of the essential amino acids (color coded) required for Jab1 binding. The change seen in this residue in the mouse S100A7/psoriasin protein is a conservative change and the residue (E) remains negatively charged. Amino acid color-coding: non-polar (yellow), neutral polar (green), negatively charged (red). Hs = homo sapiens, Mm = Mus musculus. Adapted from [6].

A putative Jab1-binding motif is also present in the murine S100A7/psoriasin sequence but is absent from the human S100A15 sequence (Figure 5). Of the three amino acid residues known to be conserved in the Jab-1 binding motif of Jab-1 binding proteins (see highlighted residues in Figure 5), one is different in the mouse S100A7/psoriasin. However, the change from aspartic acid in the human S100A7 to a glutamic acid in mouse S100A7/psoriasin is a conservative change and the residue remains acidic. In contrast, the equivalent residue in human S100A15 is threonine, a neutral residue containing an hydroxyl group in its aliphatic side chain, and also having the potential to be post-translationally modified by phosphorylation. These observations support further our hypothesis that the so-called mouse S100A15/psoriasin gene encodes a product that is functionally more similar to human psoriasin/S100A7 than human S100A15. Since the human and mouse Jab1 proteins are 99% identical (Genbank accession number, mouse Jab1 AAC33900; human Jab1 NP_00364) we speculate that conservation of the Jab1-binding domain in the mouse protein results in conservation of the interaction as well.

### Expression of mouse S100A7/psoriasin during mouse mammary tumorigenesis

Our initial results shown in Figure 2 suggest that the expression of mouse S100A7/psoriasin may be upregulated during mammary tumorigenesis. This was of interest since we and others have previously found that human psoriasin/S100A7 expression is highly up-regulated in human breast cancer compared to normal breast tissue [2,24]. To confirm and extend this observation, further analysis of murine mammary tumors was conducted by both semi-quantitative RT-PCR and by *in situ *hybridization.

cDNAs were generated from matched normal mammary gland and mammary gland tumors from DMBA-treated CD1 wild-type mice (n = 6, same as in Figure 2A). Semi-quantitative RT-PCR analysis showed significantly increased expression of mouse S100A7/psoriasin mRNA in mammary gland tumors relative to matched normal mammary gland (p < 0.05) by Wilcoxon signed-rank test, one-sided (Figure 2B).

**Figure 6:**
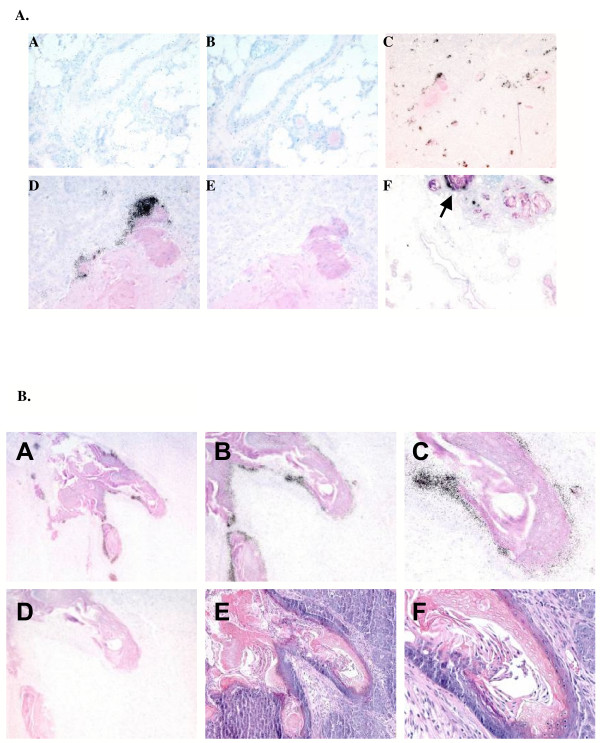
A Strong focal expression of mouse S100A7/psoriasin RNA in mammary gland tumors compared to little expression in matched normal mammary gland tissue using *in situ *hybridization Sections from formalin fixed paraffin-embedded mammary tissue samples matched to the frozen tissue from which RNA was previously isolated for RT-PCR analysis were used for *in situ *hybridization analysis of mouse S100A7/psoriasin expression: A, C, D, F show hybridization with mouse S100A7/psoriasin ^35^S-labeled antisense cRNA; B, E show hybridization with mouse S100A7/psoriasin ^35^S-labeled sense cRNA. A = high magnification (400X) section of normal mammary gland, hybridized with mouse S100A7/psoriasin ^35^S-labeled antisense cRNA. B = high magnification (400X) adjacent section to A hybridized with mouse S100A7/psoriasin ^35^S-labeled sense cRNA. C = low magnification (100X) of section from the matched DMBA mammary tumor from same animal shown in A hybridized with mouse S100A7/psoriasin ^35^S-labeled antisense cRNA. D = higher magnification (400X) of C. E = high magnification (400X) of adjacent section from the same mammary tumor shown in D, hybridized with mouse S100A7/psoriasin ^35^S-labeled sense cRNA. F = low magnification (100X) of a single tissue section (tissue from a different mouse to that shown in A-E)) hybridized with mouse S100A7/psoriasin ^35^S-labeled antisense cRNA, where strong focal expression of mouse S100A7/psoriasin RNA is observed in the mammary gland tumor (black arrow) with little or no expression in the surrounding normal mammary gland tissue. B Localization of mouse S100A7/psoriasin expression to areas of squamous differentiation in DMBA-induced mammary gland tumors Sections from formalin fixed paraffin-embedded DMBA-induced mammary tumor samples matched to the frozen tissue from which RNA was previously isolated for RT-PCR analysis were used for *in situ *hybridization analysis of mouse S100A7/psoriasin expression. A = low magnification (100X) of tumor section hybridized with mouse S100A7/psoriasin ^35^S-labeled antisense cRNA. B = higher magnification of A (200X), C = high magnification of B. D = adjacent section to that seen in A, B, C, and at same magnification as B but hybridized with mouse S100A7/psoriasin ^35^S-labeled sense cRNA. E and F are haematoxylin and eosin stains of adjacent sections to that shown in A, B, C, and D. E is the same magnification as in B, and F is the same magnification as that shown in C. Squamous differentiation in the haematoxylin and eosin stained adjacent sections is visualized as a characteristic "pearling" pattern where epithelial tumor cells (purple) differentiate into keratinocytes (pink) before denucleating and sloughing off into the inner lumen (white). Thus cells on the outer margin of the pearled area are undergoing squamous differentiation, and those correspond to cells expressing mouse S100A7/psoriasin RNA, in A, B, and C.

To confirm this differential expression and to determine which cell type within the mammary tumors was expressing mouse S100A7/psoriasin, *in situ *hybridization was performed on sections from paraffin-embedded tissue blocks corresponding to the fresh frozen samples from which RNA was extracted to generate cDNAs used for RT-PCR analysis. A strong but focal signal was observed in all six mammary gland tumors while the signal was weak or undetectable in all of the matched normal mammary gland tissues (Table 1, Figure 6). Assessment of the sections at high magnification and correlation with serial sections stained only by H&E, revealed that the S100A7/psoriasin expression was usually confined to a subset of epithelial tumor cells that showed morphological features of squamous differentiation (Figure 6). Within these regions of squamous differentiation expression was usually decreased with proximity to the squamous surface. Semi-quantitative RT-PCR and *in situ *hybridization results showed a significant positive correlation (Spearman's rank correlation coefficient r = 0.63, p < 0.02) (Table 1).

Although up-regulation of mouse S100A7/psoriasin expression occurred in DMBA induced tumors compared to adjacent normal mammary gland tissue, expression levels showed a wide-scatter amongst the mammary tumors (Figure 2). This was of interest since ERα has been reported to have variable expression in chemical carcinogen induced tumors [25-27] and we have previously shown an inverse association of human psoriasin/S100A7 expression with ERα in breast cancer [3,5]. We therefore compared the expression of mouse S100A7/psoriasin as determined by *in situ *hydridization with ERα expression assessed by IHC in adjacent sections from the same tumors. An inverse association between mouse S100A7/psoriasin and ERα expression was clearly evident but categorical contingency analysis did not reach statistical significance (Fisher's exact test, p = 0.073), likely due to small numbers. However detailed comparison within tumors showed that in tumors that expressed both genes, mouse S100A7/psoriasin expression was restricted to areas within heterogeneously positive ERα tumors that lacked ERα expression. Interestingly, mouse mammary tumors that develop due to targeting of the *neu *oncogene to the mouse mammary gland (MMTV-*neu*) did not express mouse S100A7/psoriasin RNA nor ERα (data not shown).

### Expression of mouse S100A7/psoriasin in a model of skin inflammation

**Figure 7:**
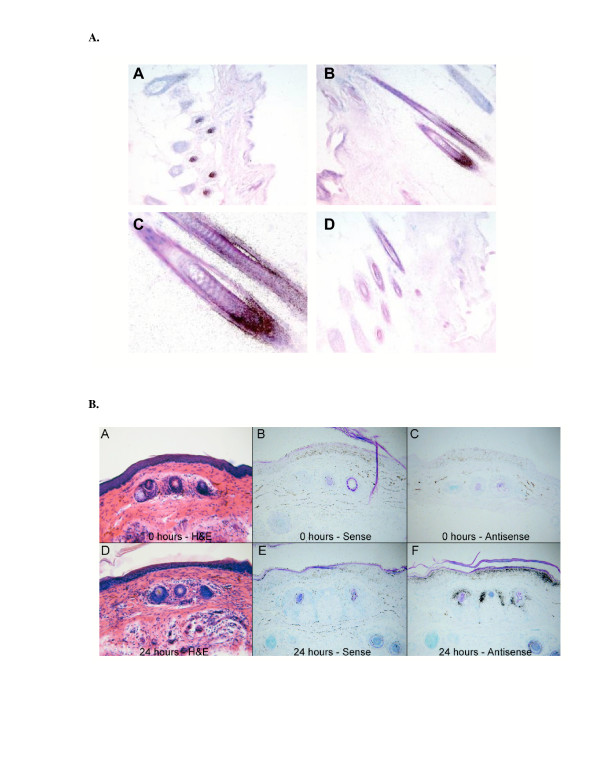
A Localization of mouse S100A7/psoriasin expression to areas of squamous differentiation in skin Sections from formalin fixed paraffin-embedded normal mouse skin samples matched to the frozen tissue from which RNA was previously isolated for RT-PCR analysis were used for *in situ *hybridization analysis of mouse S100A7/psoriasin expression. A, B, C increasing magnifications (original is 200X) of sections hybridized with mouse S100A7/psoriasin ^35^S-labeled antisense cRNA, showing strong focal expression of mouse S100A7/psoriasin RNA in a particular subset of cells surrounding the hair shafts, which are associated with squamous differentiation. D, adjacent section hybridized with mouse S100A7/psoriasin ^35^S-labeled sense cRNA. B Upregulation of mouse S100A7/psoriasin expression in a model of skin inflammation Sections from formalin fixed paraffin-embedded mouse skin tissue at 0 mins and 24 hours after croton application to the tail skin as described in Materials and Methods. A and D, haematoxylin and eosin stained sections, from 0 and 24 hr treated skin, respectively. B and E adjacent sections from 0 and 24 hr croton oil treated skin, respectively, hybridized with mouse S100A7/psoriasin ^35^S-labeled sense cRNA. C and F, adjacent sections from 0 and 24 hr croton oil treated skin, respectively, hybridized with mouse S100A7/psoriasin ^35^S-labeled antisense cRNA.

Human psoriasin/S100A7 was originally cloned from psoriatic skin, an inflammatory skin disease [1]. Mouse S100A7/psoriasin was however first identified in an expression library constructed from normal mouse skin (Genbank Acc AA792680 and AY465109) and our data have confirmed mouse S100A7/psoriasin RNA expression in normal mouse skin (see Figure 2A). To determine if mouse S100A7/psoriasin expression is upregulated in inflammation, ISH was performed on sections from normal mouse tissues (see Figure 2A) as well as tissue from a model of mouse skin inflammation. As seen in mammary tumor tissues, a strong but focal signal was observed in some samples and assessment of the sections at high magnification revealed that the mouse S100A7/psoriasin expression was confined to a subset of epithelial cells surrounding hair shafts (Figure 7A) consistent with the localization of human psoriasin/S100A7 in normal human skin [3]. No signal was observed using a mouse S100A7/psoriasin sense probe. In addition we have speculated that mouse S100A7/psoriasin expression would mimic its human counterpart and be upregulated under conditions of skin inflammation. A significant increase in mouse S100A7/psoriasin RNA expression is seen at 24 hrs after croton oil application correlating with the development of inflammation characterized by leucocytic inflammation, (Figure 7B, Table 2).

### Expression of human psoriasin/S100A7 in relation to differentiation in human tumors

**Figure 8:**
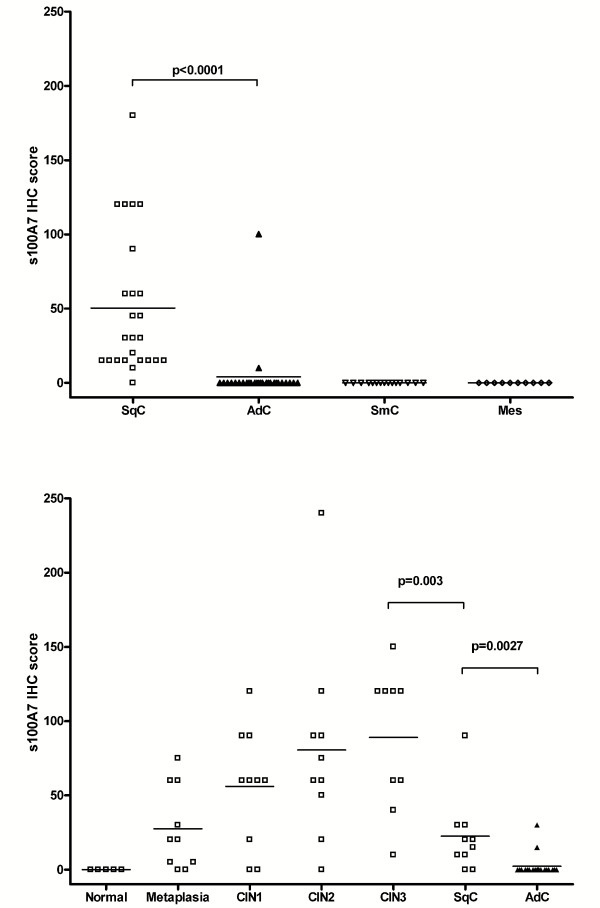
Expression of psoriasin in human lung and cervical tumors determined by immunohistochemistry Upper panel shows the scatter plot chart of level of expression (assessed by IHC score) in different lung tumor types (SqC = squamous cell carcinoma, AdC = adenocarcinoma, SmC = small cell carcinoma, Mes = mesothelioma). Bars represent the median scores. P value determined by t-test. Lower panel shows the scatter plot chart of level of expression (assessed by IHC score) in different cervical lesions corresponding to early stages of cervical tumor progression (metaplasia, CIN = cervical intra-epithelial squamous neoplasia, stages 1, 2, and 3, SqC = squamous cell carcinoma invasive, AdC = cervical adenocarcinoma, in-situ and invasive). P values determined by t-test.

The pattern of mouse S100A7/psoriasin expression observed in mouse tumors and skin suggested an association of expression with the process of squamous differentiation. Studies of human psoriasin /S100A7 support a similar association in skin and bladder cancer [28,29]. To determine further the relation between psoriasin and different pathways of epithelial differentiation we therefore examined human psoriasin expression relative to different human tumor types in the cervix and lung, since overt squamous differentiation is rare in human breast cancer. In tumors in both cervix and lung, psoriasin expression was significantly correlated with squamous differentiation (p < 0.0001). Expression was observed in over 90% of squamous carcinomas in both organs, but was a rare occurrence (<10%) in adenocarcinomas and absent in other tumor types in the lung (Figure 8). As we have previously observed in both breast and skin, psoriasin expression in the cervix was also most highly expressed in squamous carcinoma-in-situ with lower levels in invasive squamous carcinoma (p = 0.0013).

## Discussion

We have used structural and expression analysis to show that a gene currently classified as mouse S100A15 (GenBank acc. AY465110) which maps within the murine S100 cluster on chromosome 3 (see acc. NT_078386, NCBI Map View Link and LOC381493) [10] should be reclassified as mouse S100A17/psoriasin, as suggested by Marenholz *et al. *[9]. This gene is not highly conserved relative to other members of the S100 family (~40% amino acid sequence similarity of mouse S100A7 with either human S100A7 and/or human S100A15, compared to >60% amino acid sequence similarity of most other known mouse S100A proteins to their human counterparts, unpublished data) [10]. However, phylogenetic analysis shows that while mouse retained an ancestral S100A7/psoriasin gene, the human ortholog underwent duplication and functional differentiation making the assignment of orthology/paralogy only on the basis of phyogenetic information uncertain.

In the human, the closely related psoriasin/S100A7 and S100A15 appear to be functionally distinct. Although both were isolated as overexpressed genes in human psoriatic skin [1,20], we have previously identified S100A7 to be differentially expressed in neoplastic mammary gland [30]. Neither gene is substantially detectable in database analysis of the available normal human mammary gland libraries, and only psoriasin/S100A7 is present at high copy numbers (up to 2,300 tags per 200,000) in several mammary gland neoplasia libraries, whereas S100A15 is absent or rarely detectable (<10 tags per 200,000) [30]. Additionally, psoriasin/S100A7 is expressed in several SAGE database libraries representing human skin neoplasia, while S100A15 is not detected (data not shown). Thus, despite some similarities in their occurrence and expression in association with psoriasis [20] these closely related human genes show dissimilar expression patterns in mammary tumors suggesting distinct functional roles. In contrast, our studies show that the pattern of gene expression of mouse S100A7/psoriasin during mammary tumorigenesis and skin inflammation occurs in a similar pattern to that seen with human psoriasin/S100A7 [2,3]. Furthermore, while mouse S100A7/psoriasin shows approximately equivalent amino acid sequence similarity to both human S100A7 and human S100A15 proteins, both mouse S100A7/psoriasin and human psoriasin/S100A7 contain a putative Jab1-binding motif that is functionally significant in the human at least [6,7] but this motif is not found in human S100A15. We conclude on the basis of chromosomal location, phylogenetic analysis, amino acid sequence similarity, conservation of a putative Jab1-binding motif, and similarities in patterns of expression, that mouse S100A7/ psoriasin is the murine ortholog of human psoriasin/S100A7.

Expression of human psoriasin/S100A7 was originally attributed to skin pathologies where abnormal squamous differentiation occurs [1]. In breast tumors where psoriasin/S100A7 is also expressed, overt squamous differentiation is rare. However it has been detected and suggested as a marker of squamous differentiation in a subtype of bladder cancer [29]. Extending the latter finding our data here shows that in cervix and lung, two tissues where squamous and glandular differentiation are both common differentiation pathways for carcinomas, psoriasin/S100A7 is commonly expressed and almost exclusively associated with squamous tumor subtypes. The parallel finding that mouse S100A7/psoriasin was distinctively associated with areas of squamous differentiation within murine breast adenocarcinomas suggests that similar factors are involved in the regulation of both psoriasin/S100A7 and murine S100A7/psoriasin genes, and is in keeping with a similar role for the murine gene.

A role for human psoriasin/S100A7 in inflammation has been suggested, since psoriasin/S100A7 can be secreted and was shown to be chemotactic for neutrophils and CD4+ T-cells *in vitro *[31]. Our current data from a model of mouse skin inflammation is also consistent with such a role, since a significant upregulation in mouse S100A7/psoriasin expression occurs simultaneously with the acute phase of skin inflammation after application of croton oil to the mouse tail skin. The role for psoriasin/S100A7 in tumorigenesis may be related to the activity of pro-survival pathways [7] and acquisition of apoptosis resistance [8]. Our results here also provide indirect support for this hypothesis, as mouse S100A7/psoriasin RNA was expressed in keratinocytes at the margin of squamous differentiation, where resistance to apoptotic stimuli may be an important component of the sequence of differentiation and to allow time for production of large amounts of keratin before finally undergoing desquamation, denucleation and cell death [32].

Finally, psoriasin has been implicated in human breast cancer progression. Specifically, psoriasin/S100A7 has been associated with the pre-invasive DCIS phenotype [2], augmentation of several characteristics of malignancy *in vitro *and *in vivo *[4,6] and with poor outcome in invasive estrogen receptor-negative tumors [3,5]. Our current results also support the involvement of S100A7/psoriasin in murine mammary tumorigenesis. Additionally, differential expression of mouse S100A7/psoriasin was observed between DMBA and MMTV-*neu *induced mammary tumors, and a strong trend towards a negative correlation between mouse S100A7/psoriasin and estrogen receptor alpha expression emerged in the DMBA induced mammary tumors. Such data are consistent with the pattern of expression of human psoriasin/S100A7 in human breast tumors, and are consistent with the view that mouse S100A7/psoriasin subserves similar roles to human psoriasin/S100A7 in mammary tumorigenesis and breast cancer progression. It is nevertheless possible that human psoriasin/S100A7 and mouse S100A7/psoriasin have several functions, depending on cellular context. This is reflected by differences already observed in localization of expression in different cell types within a tissue or subcellular localization, since psoriasin has been found in the nucleus, in the cytoplasm, at the cell periphery/plasma membrane and it can also be secreted [33,34].

## Conclusion

S100 proteins have been known to be differentially expressed during tumorigenesis as well as other disease states for some time. However, the functional roles they may play in disease processes are poorly understood [33,34]. In breast cancer, psoriasin/S100A7 is associated with important biological and clinical aspects of the disease [30] and the identification of its potential ortholog in the mouse is an important step to facilitate understanding of its function and mechanism of action. The results presented in this study strongly support the hypothesis that mouse S100A7 is the murine ortholog of human psoriasin/S100A7, and provide a rationale for the manipulation of mouse S100A7/psoriasin in mice to gain important insights into the function of human psoriasin/S100A7.

### Competing interests

The author(s) declare that they have no competing interests.

### Authors' contributions

MW carried out the database searches, cloning, sequencing, ISH, RT-PCR of mouse S100A7/psoriasin, phylogenetic analysis and co-ordinating frozen tissue and tissue block collection from other authors. She was involved in data analysis and writing of the manuscript and critically revising it.

EE helped MW in database searches and motif analyses. He was involved in writing and revising it critically.

ML carried out the mouse skin inflammation experiments and prepared the tissue blocks. He was involved in writing and revising it critically.

SA, GQ and AA were involved in carrying out the tissue analysis and data acquisition and analysis in Figure 8.

LS-C supervised and carried out some of the ISH.

YN carried out the IHC.

AC supervised and helped carry out the phylogenetic analysis. He was involved in writing the manuscript and revising it critically.

YM and RS provided the mouse mammary tumor tissues, other mouse tissues and respective blocks for analysis. They were both involved in writing the manuscript and revising it critically.

LCM is the Principal Investigator of the project and was responsible for the overall supervision and co-ordination of the project. She is the corresponding author and contributed to the writing, critical revision together with draft preparation, final preparation for manuscript submission, preparation and submission of all revisions.

PHW is the co-P.I. of the project. He supervised and was involved in the interpretation of all histopathology, ISH and IHC. He contributed to all data analysis, to the writing of the manuscript and its critical revision.

## Pre-publication history

The pre-publication history for this paper can be accessed here:



## Figures and Tables

**Table 1:** Correlation of mouse S100A7/psoriasin expression using RT-PCR and *in situ *hydridization in normal mammary gland and DMBA-induced mammary tumors Mouse S100A7/psoriasin mRNA levels were determined in extracts from frozen tissue samples of matched normal mammary gland and DMBA-induced mammary tumors (n = 6) by semi-quantitative RT-PCR as described in Materials and Methods. Sections from the corresponding adjacent tissue samples which had been formalin fixed and paraffin-embedded, where used for *in situ *hybridization (ISH) with ^35^S-labelled antisense mouse S100A7/psoriasin probes. Specific hybridization was scored on a scale of 0 to 3 as described in Materials and Methods. Results of the two analyses were correlated using Spearmans test (r = 0.63, p < 0.02).

		Relative mouse S100A7/psoriasin expression	
	Sample	RT-PCR	ISH

Normal MG	1	0.37	0
	2	0.16	0
	3	0.14	0
	4	0.21	<0.5
	5	0.73	<0.5
	6	0.42	0

MG Tumor	1	1.50	2
	2	1.63	3
	3	1.12	2
	4	0.22	1
	5	1.22	1
	6	0.13	1

**Table 2:** Inflammatory response to topical application of croton oil to the skin.

	0 hours median [range]	24 hours median [range]
Experiment #1	9 [3-21]	15 [4-32]***
Experiment #2	10 [0-19]	20 [10-52]***
Experiment #3	13 [0-24]	25 [9-48]***

The number of nuclei per 50 μm^2 ^area of dermis was used as a parameter to evaluate inflammation.

Note – in all three experiments, the medians were found to be significantly different from each other (P < 0.0001) using the Mann-Whitney U test.
